# Docosahexaenoic acid alleviates DSS-induced colitis by regulating the gut microbiota and restoring the gut barrier

**DOI:** 10.1007/s10123-026-00859-4

**Published:** 2026-07-03

**Authors:** Sumin Wang, Shiping Hu, Yan Yan, Lingyi Wu, Li Tang, Yiyang Hu, Wanneng Wang

**Affiliations:** 1 Department of Pharmacy, College of Pharmaceutical and Biological Engineering, Chongqing University of Technology, Chongqing, 400054 China; 2 Department of Gastroenterology, Xinqiao Hospital, Army Medical University, Chongqing, 400037 China; 3 Department of Information Management, Xinqiao Hospital, Army Medical University, Chongqing, 400037 China; 4 Department of Oncology, The General Hospital of Western Theater Command, Chengdu, 610036 Sichuan China

**Keywords:** Docosahexaenoic acid, DSS-induced colitis, Gut microbiota, Intestinal barrier, Fecal microbiota transplantation, Regulatory T cells

## Abstract

Docosahexaenoic acid (DHA), a long-chain omega-3 polyunsaturated fatty acid, has well-recognized anti-inflammatory activity; however, the mechanisms underlying its protective effects in inflammatory bowel disease (IBD) remain incompletely understood. In this study, we investigated the effects of DHA in a dextran sulfate sodium (DSS)-induced mouse model of colitis and examined whether these effects were mediated by the gut microbiota. DHA administration markedly alleviated DSS-induced colitis, as indicated by reduced body weight loss, disease activity, mortality, colon shortening, histological injury, intestinal barrier disruption, and colonic inflammatory responses. 16 S rRNA gene sequencing showed that DHA reshaped the gut microbial community and increased the abundance of beneficial taxa, including *Bifidobacterium*. Antibiotic cocktail (ABX)-mediated microbiota depletion largely abolished the protective effects of DHA, whereas fecal microbiota transplantation (FMT) from DHA-treated donors transferred resistance to DSS-induced colitis to recipient mice. DHA also restored tight junction protein expression and increased the frequency of colonic regulatory T cells in a microbiota-dependent manner. These findings indicate that DHA alleviates experimental colitis by modulating the gut microbiota, restoring intestinal barrier integrity, and regulating mucosal immune homeostasis. DHA may therefore represent a promising dietary strategy for the prevention or adjunctive treatment of ulcerative colitis (UC).

**Supplementary Information:**

The online version contains supplementary material available at https://doi.org/10.1007/s10123-026-00859-4.

## Introduction

Inflammatory bowel disease (IBD) comprises chronic relapsing inflammatory disorders of the gastrointestinal tract, mainly ulcerative colitis (UC) and Crohn’s disease (CD). The global incidence and prevalence of IBD have increased substantially, particularly in newly industrialized and developing regions (Ng et al. 2017). Although several pharmacological approaches are available for IBD, many current treatments are limited by incomplete efficacy, high cost, and potential adverse effects (Luo et al. 2022). Therefore, safe nutritional or dietary interventions that can complement existing therapies remain of considerable interest for IBD management.

UC is a multifactorial disease driven by interactions among genetic susceptibility, environmental factors, intestinal microbes, mucosal barrier dysfunction, and dysregulated immune responses (Kobayashi et al. 2020). Accumulating evidence indicates that gut microbiota dysbiosis is closely associated with IBD pathogenesis (Shan et al. 2022). Compared with healthy individuals, patients with IBD often show reduced microbial diversity and compositional imbalance, including increased abundances of Proteobacteria and Actinobacteria and decreased abundances of Firmicutes and Bacteroidetes (Halfvarson et al. 2017). Fecal microbiota transplantation (FMT) has also been explored as a therapeutic strategy for patients with active or recurrent intestinal inflammation (Gupta and Khanna 2017; Sood et al. 2020). These observations suggest that targeting the intestinal microbiota may be a promising strategy for UC prevention and treatment.

The intestinal mucosal barrier consists of mechanical, immune, biological, and chemical components that act cooperatively to limit the invasion of pathogens and luminal antigens (Paone and Cani 2020). Barrier impairment is a hallmark of UC (Salim and Soderholm 2011). Microbial dysbiosis can disrupt intestinal immune homeostasis, impair epithelial repair, and promote inflammatory responses. Aberrant activation of innate and adaptive immunity is a characteristic feature of UC pathology (Geremia et al. 2014). Activated immune cells produce pro-inflammatory mediators, including tumor necrosis factor-α (TNF-α), interleukin (IL)-17 A, and other cytokines, which further damage the epithelial barrier and aggravate disease progression (Jiang et al. 2023). In contrast, regulatory T (Treg) cells contribute to mucosal tolerance and intestinal homeostasis partly through the production of anti-inflammatory mediators such as IL-10 (Clough et al. 2020).

Docosahexaenoic acid (DHA) is an omega-3 (n-3) polyunsaturated fatty acid (PUFA) that must be obtained largely from dietary sources because endogenous synthesis is limited in mammals (Hashimoto et al. 2017). Previous studies have shown that DHA supplementation may benefit cognitive decline in elderly individuals and patients with Alzheimer’s disease (Chu et al. 2022; Weiser et al. 2016). DHA also has anti-inflammatory properties and has been investigated in inflammation-associated disorders, including rheumatoid arthritis, cancer, cardiovascular disease, and IBD (Dawczynski et al. 2018; Marton et al. 2019; Yamagata 2017; Yum et al. 2016). Epidemiological and clinical evidence further suggests an inverse relationship between intake of n-3 PUFAs and the risk or severity of IBD (MacLean et al. 2005). Nevertheless, the precise mechanisms by which DHA modulates intestinal inflammation remain incompletely understood.

The present study aimed to determine whether DHA alleviates DSS-induced colitis and to clarify the contribution of the gut microbiota, intestinal barrier function, and mucosal immune regulation to its protective effects. Our findings provide evidence that DHA mitigates experimental colitis through microbiota-dependent mechanisms and may have potential as a dietary strategy for UC management.

## Materials and methods

### Dextran sulfate sodium (DSS)-induced colitis

After a 7-day acclimatization period, eight-week-old male C57BL/6 mice weighing 20–22 g were randomly assigned to four groups: control, DSS model, DHA + DSS, and sulfasalazine (SASP, positive control) + DSS. Colitis was induced using a previously described protocol with minor modifications (Huang et al. 2022). Briefly, mice were given 2.5% DSS (MP Biomedicals, UK) in drinking water for 7 consecutive days, followed by normal drinking water for 5 days. Mice in the DHA + DSS group received DHA (35 mg/kg), suspended in corn oil, by oral gavage from the beginning of the experiment until the end of the study. Mice in the SASP + DSS group received SASP at 100 mg/kg by oral gavage once daily throughout the experiment. Disease activity index (DAI)-related parameters, including body weight loss, stool consistency, and hematochezia, were monitored and recorded daily. On day 12, mice were humanely euthanized, colon length was measured, and fecal samples were collected for subsequent analysis of the gut microbiota.

### Antibiotic cocktail (ABX) experiments and fecal microbiota transplantation (FMT)

To deplete the intestinal microbiota, mice received a broad-spectrum antibiotic cocktail (ABX) consisting of ampicillin (200 mg/kg), neomycin sulfate (200 mg/kg), metronidazole (200 mg/kg), and vancomycin (100 mg/kg) by oral gavage once daily for 5 days before DSS treatment. On day 6, colitis was induced as described above. All antibiotics were obtained from Sangon Biotech Co., Ltd. (Shanghai, China). FMT was performed using a modified version of a previously described method. Briefly, colitis was induced in donor mice that received either vehicle or DHA as described above. Recipient mice were treated with ABX for 5 days to deplete the gut microbiota. Fresh fecal samples from donor mice were then collected, suspended in phosphate-buffered saline (PBS), and administered to recipient mice by oral gavage once daily for 5 days.

### Histopathology

Distal colon samples were collected and fixed in 10% neutral-buffered formalin for 24 h. After fixation, samples were embedded in paraffin, sectioned at 5 μm, and stained with hematoxylin and eosin (H&E). Histological scores were determined according to established criteria (Chassaing et al. 2014), including the extent of epithelial damage and the depth of inflammatory cell infiltration.

### FITC-dextran intestinal permeability assay

Intestinal permeability was assessed using fluorescein isothiocyanate (FITC)-dextran as previously described (Dong et al. 2022). Briefly, mice were fasted for 3 h and then orally administered FITC-dextran (Sigma-Aldrich, St. Louis, MO, USA) at 0.6 mg/g body weight. Serum samples were collected 4 h later. Fluorescence was measured at an excitation wavelength of 485 nm and an emission wavelength of 528 nm using a Varioskan Flash Multimode Reader (Thermo Fisher Scientific, Waltham, MA, USA).

### RNA extraction and quantitative real-time PCR

Total RNA was extracted from colon tissues using RNAiso Plus reagent (Takara Biotechnology, Dalian, China). Complementary DNA (cDNA) was synthesized from 1 µg of RNA using a PrimeScript RT reagent kit (Takara, Japan). Quantitative real-time polymerase chain reaction (qRT-PCR) was performed on an ABI 7300 HT Fast Real-Time Cycler (Applied Biosystems, Foster City, CA, USA) using a TB Green Premix Ex Taq II kit (Takara, Japan). The mRNA expression levels of target genes were normalized to Actin and calculated using the comparative Ct method. Primers used for qRT-PCR were synthesized by Sangon Biotech Co., Ltd. (Shanghai, China), and their sequences are listed in Supplementary Table 1.

### Western blotting

Colon tissue samples were homogenized in radioimmunoprecipitation assay (RIPA) buffer supplemented with phenylmethylsulfonyl fluoride (PMSF) protease inhibitor. Protein concentrations were determined using a bicinchoninic acid (BCA) protein assay kit according to the manufacturer’s protocol (Beyotime Biotechnology, Shanghai, China). Equal amounts of protein were separated by 10% sodium dodecyl sulfate-polyacrylamide gel electrophoresis (SDS-PAGE) and transferred to Immobilon polyvinylidene difluoride (PVDF) membranes. Membranes were blocked with 5% non-fat dry milk in Tris-buffered saline containing 0.1% Tween 20 (TBST) for 1 h at room temperature and then incubated overnight at 4 °C with primary antibodies against ZO-1, occludin, or beta-actin at a dilution of 1:1000. After washing, membranes were incubated with horseradish peroxidase (HRP)-conjugated secondary antibodies for 1 h at room temperature. Protein bands were visualized using enhanced chemiluminescence (ECL) substrate (Thermo Fisher Scientific, Waltham, MA, USA).

### DNA extraction and 16 S rRNA gene sequencing

Total DNA was extracted from fecal samples using a TIANamp stool DNA kit (TIANGEN, Beijing, China). The quality and concentration of extracted DNA were assessed using a NanoDrop 2000 C spectrophotometer (Thermo Fisher Scientific, Waltham, MA, USA) and 1.0% agarose gel electrophoresis. The V3-V4 hypervariable regions of the bacterial 16 S rRNA gene were amplified using the primers 341 F (5’-ACTCCTACGGGRSGCAGCAG-3’) and 806R (5’-GGACTACVVGGGTATCTAATC-3’). After purification with a GeneJET gel extraction kit (Thermo Fisher Scientific, Waltham, MA, USA), PCR products were sequenced on an Illumina NovaSeq 6000 platform (Novogene, Tianjin, China).

### Immunohistochemistry

Immunohistochemical staining was performed according to a previously published protocol (Zhao et al. 2020). Briefly, 5-µm sections of paraffin-embedded colon tissue were deparaffinized in xylene and rehydrated through graded ethanol. Antigen retrieval was performed by heating sections in 10 mM sodium citrate buffer (pH 6.0) in a microwave for 10 min, followed by cooling to room temperature. Sections were then blocked with 3% hydrogen peroxide and goat serum and incubated overnight at 4 °C with primary antibodies against occludin (Santa Cruz Biotechnology, Santa Cruz, CA, USA) or zonula occludens-1 (ZO-1; Santa Cruz Biotechnology). After washing three times with PBS for 5 min each, sections were stained with 3,3’-diaminobenzidine (DAB) and examined under a microscope.

### Bioinformatic analysis

Raw sequencing data were analyzed using QIIME2 (version 2020.2). Reads were denoised and quality filtered using the DADA2 pipeline. Representative sequences were classified using the classify-sklearn algorithm against the Greengenes database (version 13.8), and operational taxonomic units (OTUs) were assigned at 97% sequence similarity. The Chao1, Shannon, and ACE indices were used to evaluate alpha diversity, whereas weighted and unweighted UniFrac distances were used to assess beta diversity.

### Flow cytometric analysis

Mouse colons were isolated, longitudinally opened, and washed with cold PBS. The tissues were incubated in 10 mL Hank’s balanced salt solution (HBSS; Lonza, Basel, Switzerland) supplemented with 1 mM dithiothreitol (DTT) and 5 mM ethylenediaminetetraacetic acid (EDTA) at 37 °C for 30 min with shaking at 300 rpm. After washing, colons were minced and digested in 15 mL HBSS containing 10% fetal bovine serum (FBS), 1.5 mg/mL type VI collagenase, and 40 µg/mL DNase I at 37 °C for 40 min with continuous shaking at 300 rpm. Single-cell suspensions were passed through 70-µm cell strainers and centrifuged at 1300 rpm for 5 min at 4 °C. Cells were stained with a live/dead viability dye and antibodies, including PerCP-Cy5.5-conjugated anti-mouse CD45, Brilliant Violet 510-conjugated anti-mouse CD4, PE-conjugated anti-mouse CD25, and AF647-conjugated anti-mouse Foxp3, according to the manufacturer’s instructions. Samples were analyzed using a Beckman Gallios flow cytometer (Beckman Coulter, Brea, CA, USA), and data were analyzed using FlowJo software.

### Statistical analysis

Data are presented as the mean ± standard deviation (SD) unless otherwise indicated. Comparisons between two groups were performed using unpaired Student’s t tests. Comparisons among multiple groups were performed using one-way analysis of variance (ANOVA) followed by Tukey’s multiple-comparison test. *P* < 0.05 was considered statistically significant. NS indicates not significant.

## Results

### DHA administration significantly alleviates DSS-induced colitis

**Fig. 1:**
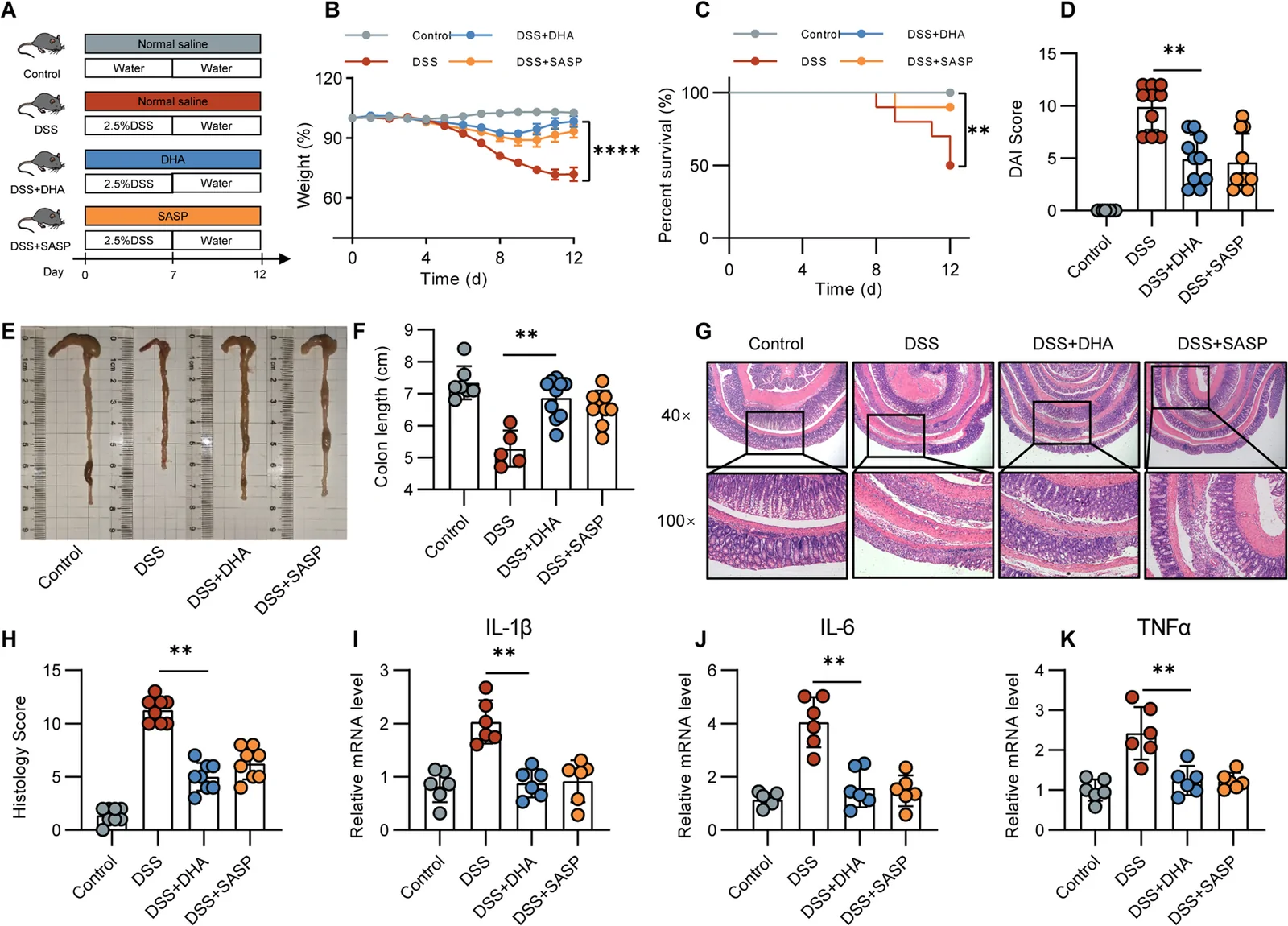
DHA supplementation ameliorates DSS-induced colitis. (A) Schematic of the experimental design. (B) Body weight changes. (C) Survival curve. (D) Disease activity index (DAI) scores. (E) Representative images of colon length measurements. (F) Quantification of colon length. (G) Representative images of hematoxylin and eosin (H&E)-stained colon sections (40x and 100x magnification). (H) Histological scores. (I-K) mRNA expression of IL-1β, IL-6, and TNF-α. Data are presented as mean ± SD (*n* = 8 mice/group). P values were calculated using two-way ANOVA. *P < 0.05, **P < 0.01

To evaluate the anti-inflammatory effects of DHA, we established an acute DSS-induced colitis mouse model that reproduces several clinical and pathological features of human UC (Fig. 1A). During model development, DSS-treated mice exhibited progressive body weight loss, diarrhea, and bloody or mucous stools. DHA administration markedly alleviated DSS-induced colitis, as shown by reduced body weight loss (Fig. 1B), improved survival (Fig. 1C), and lower DAI scores (Fig. 1D). DHA also attenuated DSS-induced colon shortening (Fig. 1E, F). Histopathological examination showed epithelial injury, disruption of glandular architecture, loss of goblet cells, and marked inflammatory cell infiltration in the DSS group, whereas these pathological changes were alleviated by DHA treatment (Fig. 1G, H). In addition, DHA reduced the colonic mRNA expression of pro-inflammatory cytokines, including IL-1β, IL-6, and TNF-α (Fig. 1I-K). These findings indicate that DHA supplementation effectively alleviates DSS-induced experimental colitis.

### The protective effect of DHA is dependent on the gut microbiota

**Fig. 2:**
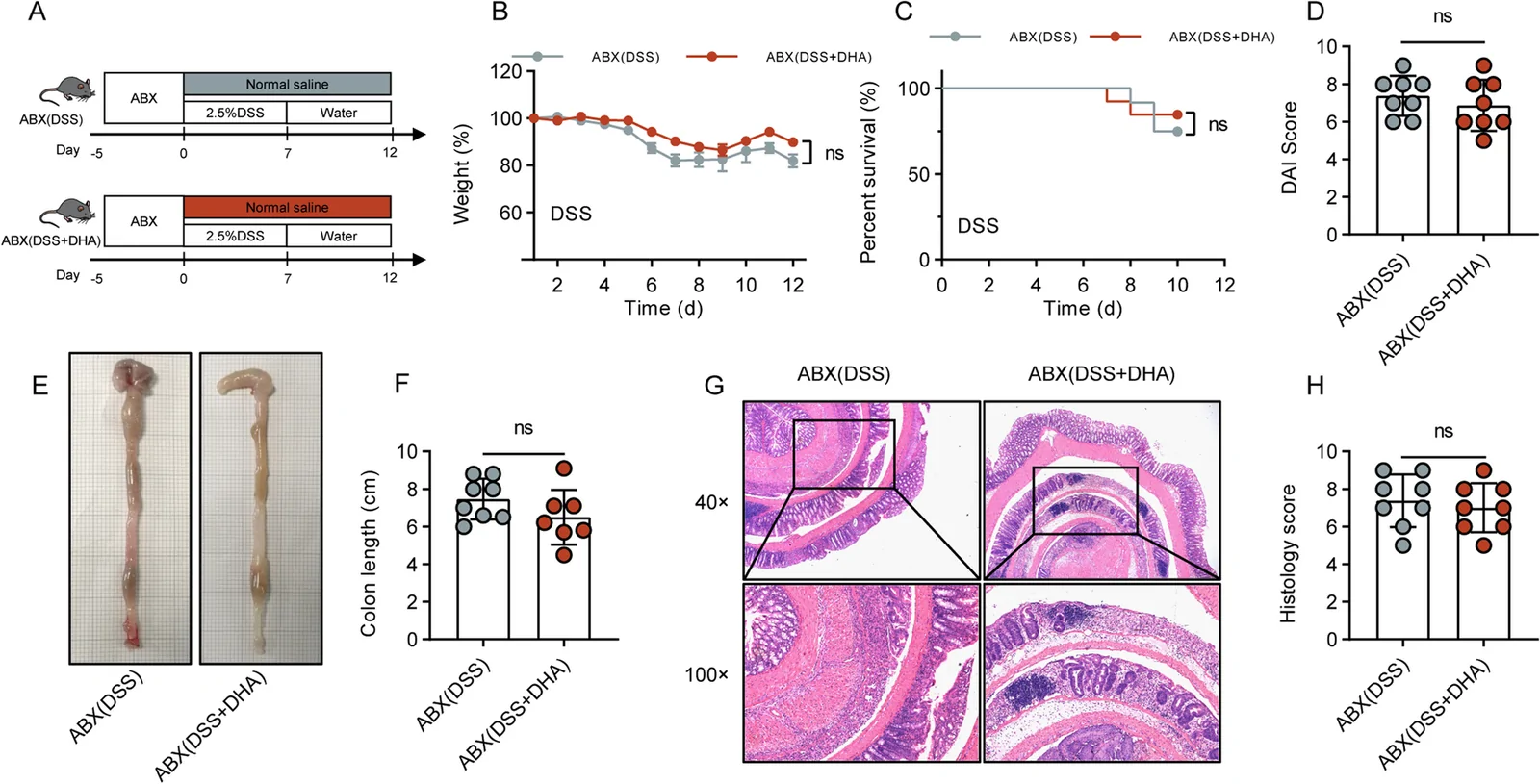
The protective effects of DHA against DSS-induced colitis disappear after gut microbiota depletion. (A) Schematic of the ABX experiment. (B) Body weight changes. (C) Survival curve. (D) DAI scores. (E) Representative images of colon length measurements. (F) Quantification of colon length. (G) Representative H&E-stained colon sections (40x and 100x magnification). (H) Histological scores. Data are presented as mean ± SD (*n* = 8 mice/group). P values were calculated using an unpaired t test. *P < 0.05, **P < 0.01

Because intestinal microbiota dysbiosis is closely associated with the development and progression of UC, we next examined whether the gut microbiota was required for the protective effect of DHA. The intestinal microbiota was depleted by ABX treatment before DSS exposure (Fig. 2A). Microbiota depletion markedly attenuated the beneficial effect of DHA. In ABX-treated mice, body weight loss (Fig. 2B), survival (Fig. 2C), DAI scores (Fig. 2D), colon length (Fig. 2E, F), and histological injury (Fig. 2G, H) were comparable between the ABX(DSS) and ABX(DSS + DHA) groups. These results suggest that the protective effect of DHA against DSS-induced colitis requires the presence of the resident gut microbiota.

### DHA-induced anti-colitic effects are transferable through the gut microbiota

**Fig. 3:**
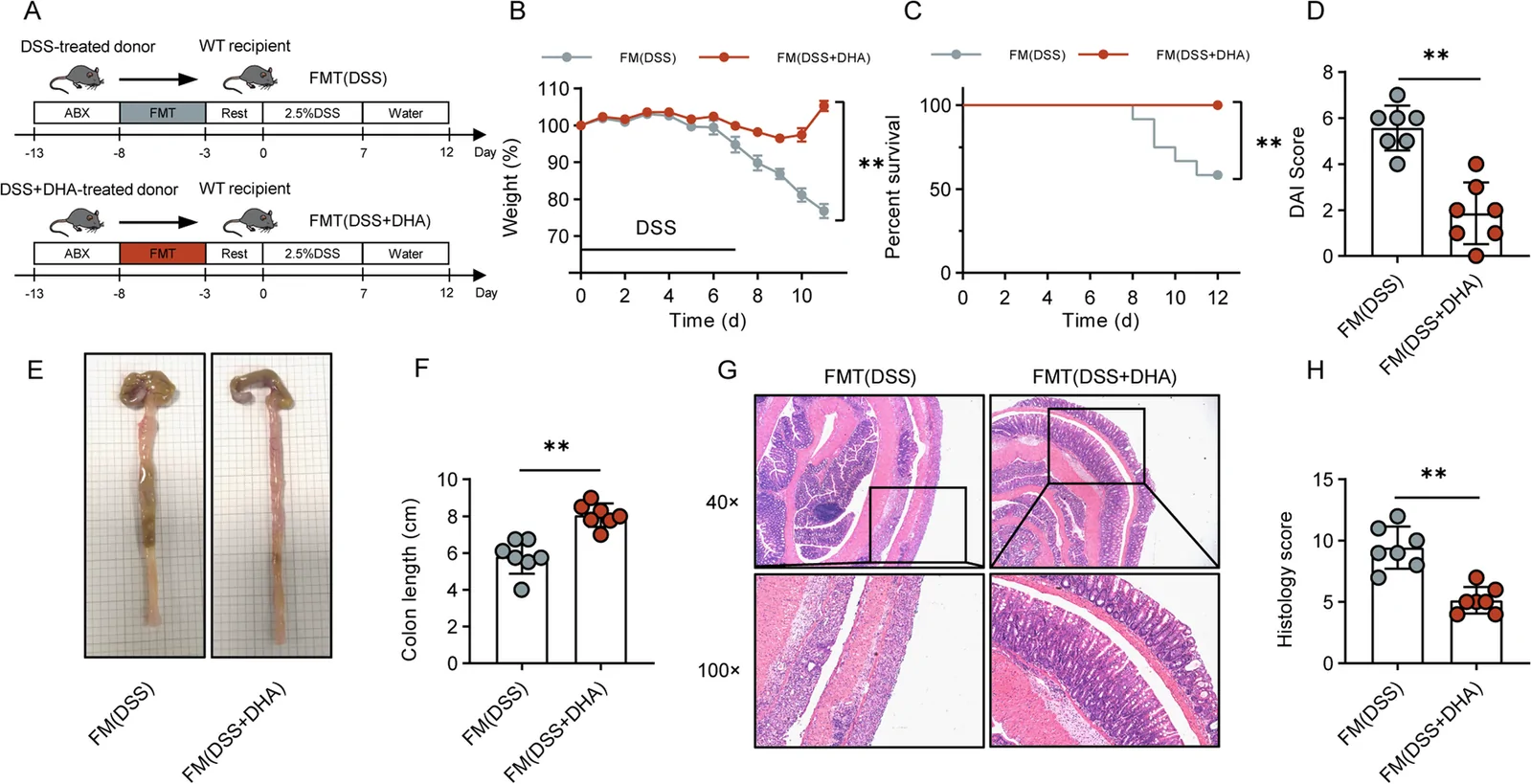
Fecal microbiota transplantation mitigates DSS-induced experimental colitis. (A) Schematic of the FMT experiment. (B) Body weight changes. (C) Survival curve. (D) DAI scores. (E) Representative images of colon length measurements. (F) Quantification of colon length. (G) Representative H&E-stained colon sections (40x and 100x magnification). (H) Histological scores. Data are presented as mean ± SD (*n* = 7 mice/group). P values were calculated using an unpaired t test. *P < 0.05, **P < 0.01, ***P < 0.001

To further determine whether DHA-mediated protection was associated with alterations in the gut microbiota, we performed FMT experiments. Microbiota-depleted recipient mice received fecal microbiota from either DSS-treated donors [FMT(DSS)] or DHA-treated DSS-challenged donors [FMT(DSS + DHA)] (Fig. 3A). Recipients of microbiota from DHA-treated donors showed less severe colitis than recipients of microbiota from DSS-treated donors. Specifically, the FMT(DSS + DHA) group exhibited reduced body weight loss (Fig. 3B), improved survival (Fig. 3C), lower DAI scores (Fig. 3D), and longer colons (Fig. 3E, F). Histological analysis also revealed reduced inflammatory infiltration, less tissue damage, and lower histological scores in the FMT(DSS + DHA) group (Fig. 3G, H). These findings indicate that DHA-induced alterations in the gut microbiota are sufficient to confer protection against DSS-induced colitis.

### DHA administration modulates gut microbiota diversity and composition

**Fig. 4:**
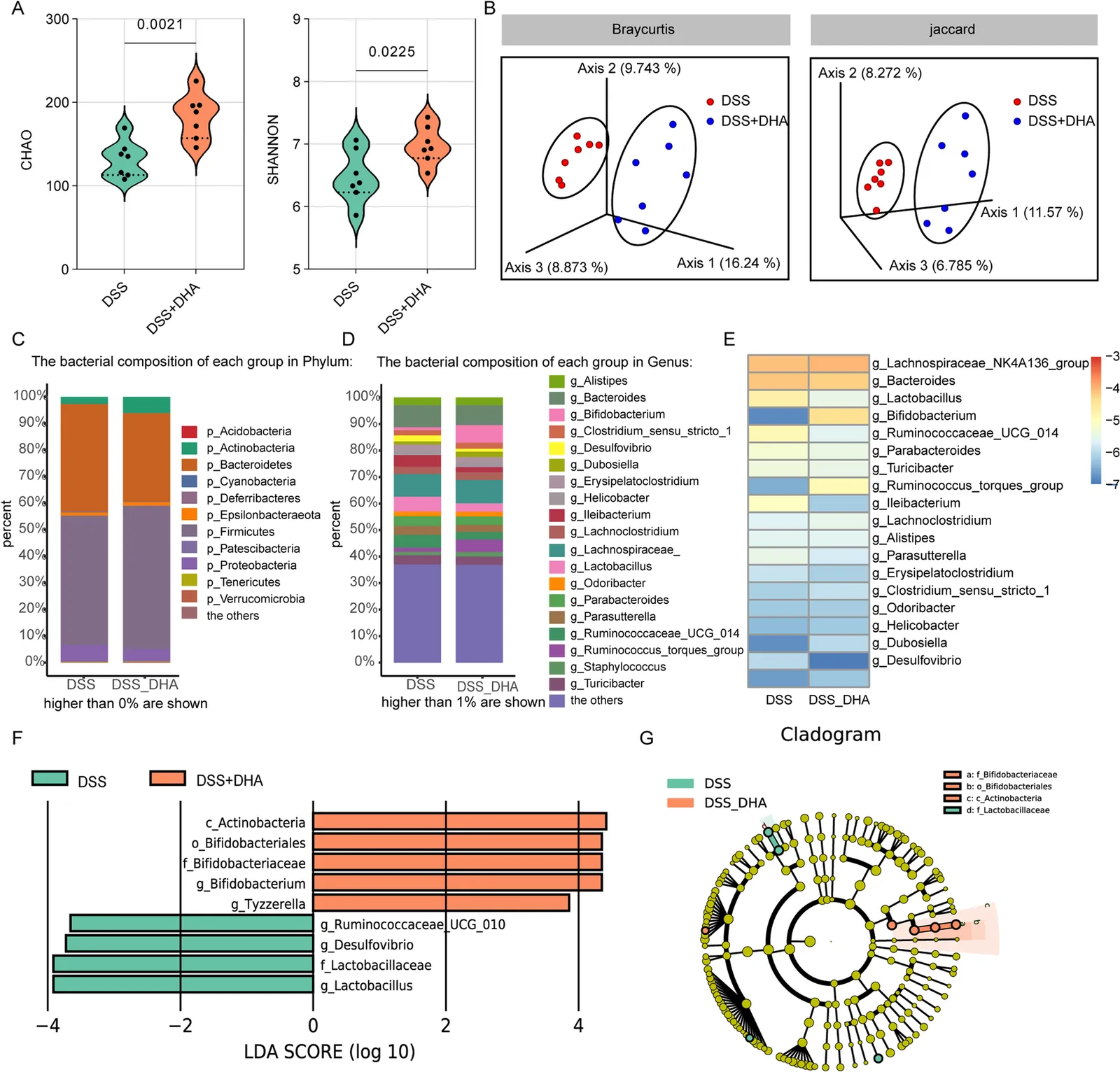
DHA treatment alters the diversity and composition of the gut microbiota. (A) Alpha diversity analysis. (B) Beta diversity analysis. (C) Relative abundance of bacterial phyla in the DSS and DSS + DHA groups. (D) Relative abundance of bacterial genera in the DSS and DSS + DHA groups. (E) Heatmap showing selected differentially abundant genera. (F) Linear discriminant analysis (LDA) score plot of differentially abundant taxonomic features between the DSS and DSS + DHA groups (LDA score > 3.5). (G) Taxonomic cladogram generated by LEfSe analysis. Green indicates taxa enriched in the DSS group; orange indicates taxa enriched in the DSS + DHA group

To characterize the effect of DHA on gut microbial composition, we performed high-throughput 16 S rRNA gene sequencing of fecal bacterial DNA from DSS and DSS + DHA mice. Alpha diversity analysis showed that DHA treatment altered microbial richness and diversity (Fig. 4A). Principal coordinate analysis (PCoA) based on Bray-Curtis and Jaccard distances revealed a clear separation of microbial community structure between the DSS and DSS + DHA groups (Fig. 4B). Taxonomic analysis at the phylum level showed that Bacteroidetes and Firmicutes were the dominant phyla in both groups, whereas the Bacteroidetes-to-Firmicutes ratio was increased in the DSS + DHA group (Fig. 4C). At the genus level, DHA treatment increased the relative abundances of *Bifidobacterium* and *Ruminococcus_torques_group* and decreased the relative abundances of *Ileibacterium* and *Ruminococcaceae_UCG_014* (Fig. 4D). Heatmap analysis further showed enrichment of *Bifidobacterium* in the DSS + DHA group and enrichment of *Ruminococcaceae_UCG_014* in the DSS group (Fig. 4E). Linear discriminant analysis effect size (LEfSe) analysis identified *Ruminococcaceae_UCG_010*, *Desulfovibrio*, and *Lactobacillus* as taxa enriched in DSS-treated mice, whereas *Bifidobacterium* and *Tyzzerella* were enriched in DHA-treated mice (Fig. 4F, G). These data demonstrate that DHA reshapes the diversity and composition of the gut microbiota during DSS-induced colitis.

### DHA ameliorates DSS-induced intestinal barrier dysfunction in a microbiota-dependent manner

**Fig. 5:**
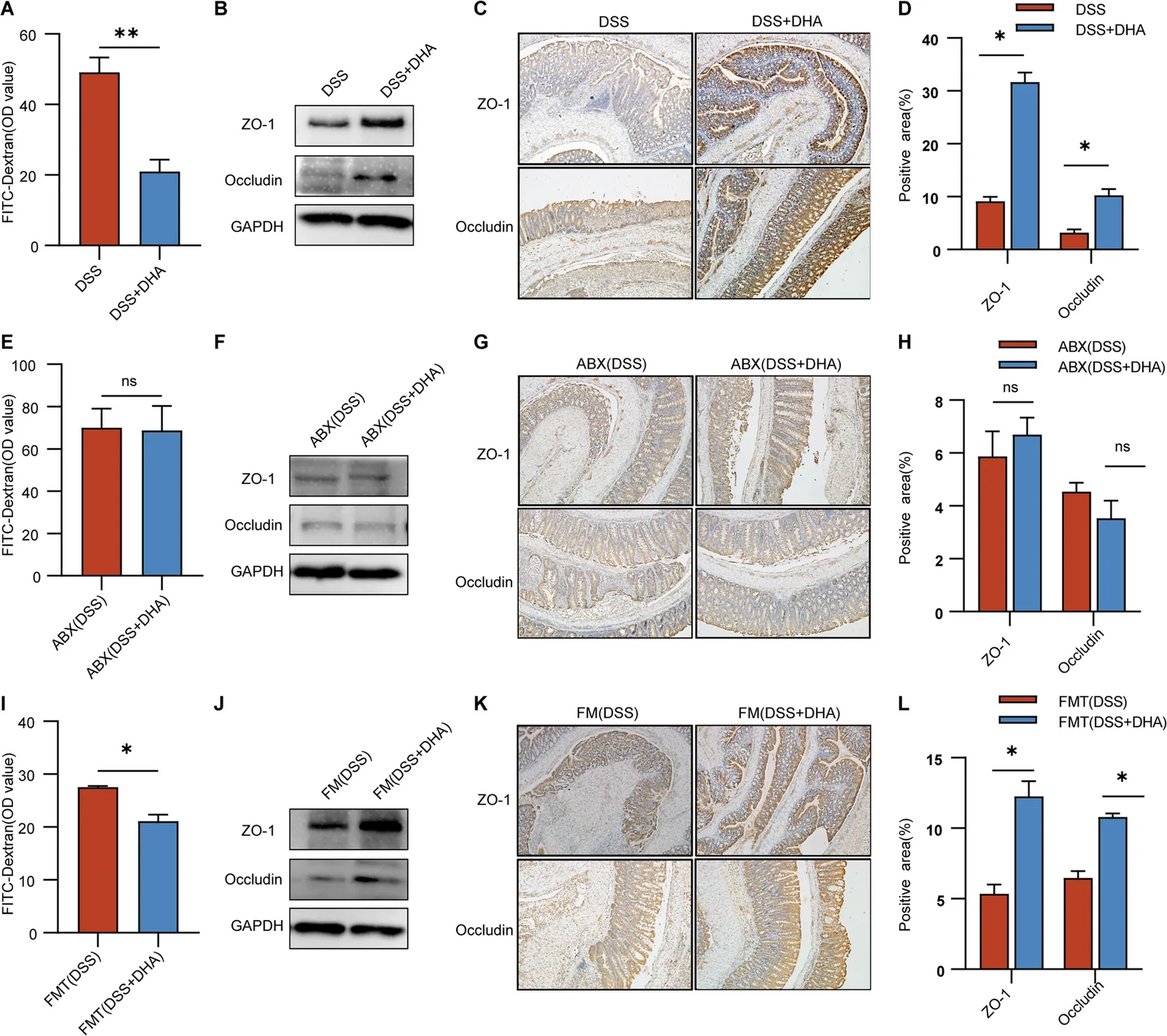
DHA improves DSS-induced intestinal barrier disruption by targeting the gut microbiota. (A) Serum FITC-dextran concentrations in DSS and DSS + DHA mice. (B) Western blot analysis of colonic occludin and ZO-1 protein levels. (C) Representative immunohistochemical staining images for ZO-1 and occludin in colon samples from DSS and DSS + DHA mice. (D) Quantification of positive staining based on integrated optical density using ImageJ. (E) Serum FITC-dextran concentrations in ABX-treated mice. (F) Western blot analysis of colonic occludin and ZO-1 protein levels in ABX-treated mice. (G) Representative immunohistochemical staining images for ZO-1 and occludin in colon samples from ABX(DSS) and ABX(DSS + DHA) mice. (H) Quantification of positive staining. (I) Serum FITC-dextran concentrations in FMT recipient mice. (J) Western blot analysis of colonic occludin and ZO-1 protein levels in FMT recipient mice. (K) Representative immunohistochemical staining images for ZO-1 and occludin in colon samples from FMT(DSS) and FMT(DSS + DHA) mice. (L) Quantification of positive staining. Data are presented as mean ± SD (*n* = 3 mice/group). P values were calculated using an unpaired t test. *P < 0.05, **P < 0.01, ***P < 0.001

Disruption of the gut microbiota can impair intestinal barrier function and promote mucosal inflammation. We therefore evaluated whether DHA affected intestinal barrier integrity. Serum FITC-dextran levels were significantly higher in DSS-treated mice than in DHA-treated mice, indicating that DHA reduced DSS-induced intestinal permeability (Fig. 5A). Consistently, DHA increased the colonic protein levels of the tight junction markers occludin and ZO-1 (Fig. 5B). Immunohistochemistry confirmed stronger expression and improved localization of ZO-1 and occludin in the DSS + DHA group compared with the DSS group (Fig. 5C, D). These findings indicate that DHA restores intestinal barrier integrity in DSS-induced colitis.

To determine whether the gut microbiota was required for the barrier-protective effect of DHA, we assessed intestinal permeability and tight junction protein expression in ABX and FMT experiments. In ABX-treated mice, DHA failed to improve intestinal permeability or restore epithelial tight junction protein expression (Fig. 5E-H). In contrast, FMT from DHA-treated donors restored intestinal barrier integrity in recipient mice with colitis, as shown by reduced intestinal permeability and increased tight junction protein expression (Fig. 5I-L). These results support an essential role for the gut microbiota in mediating the beneficial effects of DHA on intestinal barrier function.

### DHA increases colonic Treg frequency in a microbiota-dependent manner

**Fig. 6:**
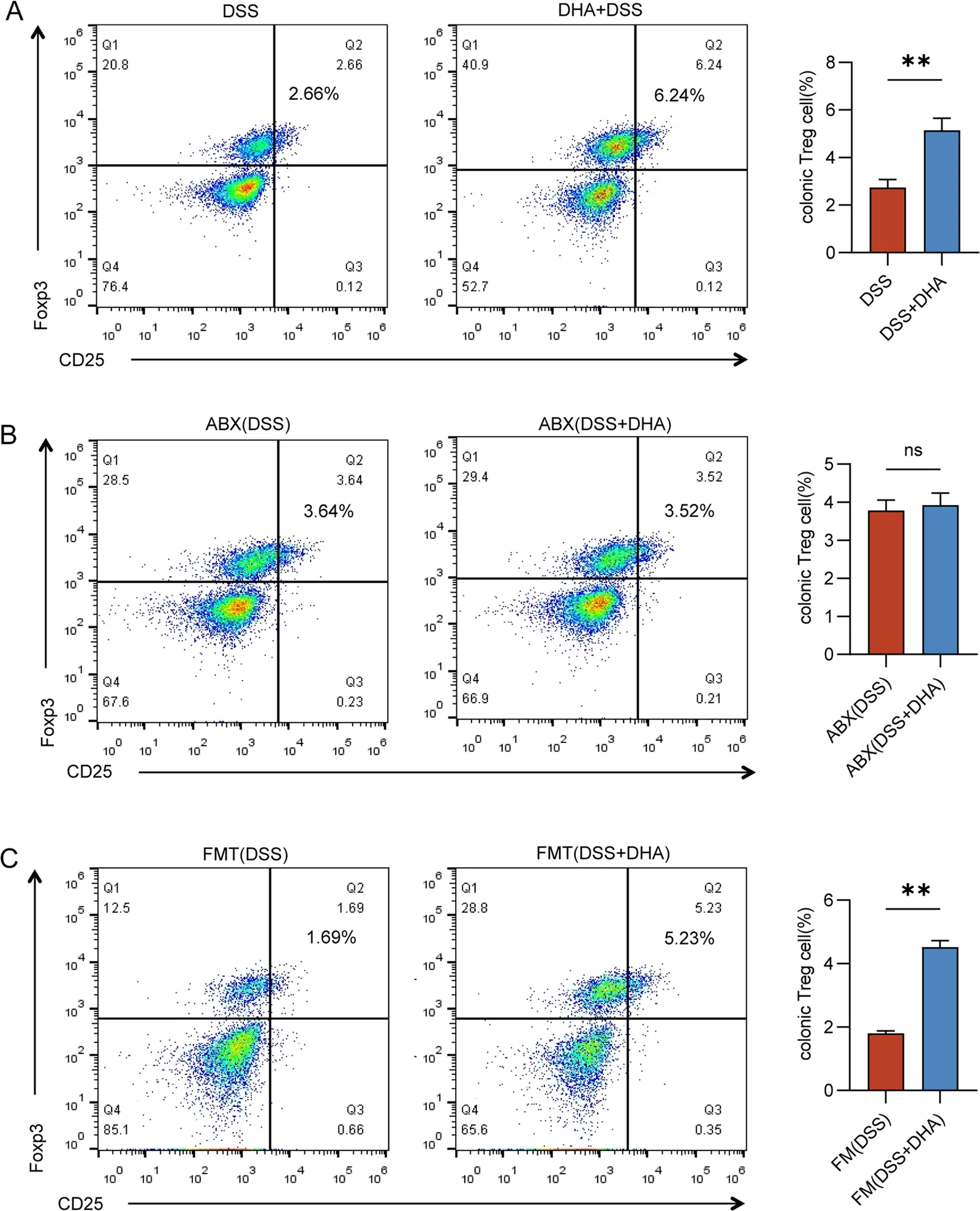
DHA treatment increases the frequency of Treg cells in DSS-induced colitis in a microbiota-dependent manner. (A) Representative flow cytometry plots of Treg cells (CD25 + Foxp3+CD4+) in the colonic lamina propria (LP) from DSS and DSS + DHA mice, with quantification of Treg cell percentages. (B) Representative flow cytometry plots and quantification of Treg cells in colonic LP from ABX(DSS) and ABX(DSS + DHA) mice. (C) Representative flow cytometry plots and quantification of Treg cells in colonic LP from FMT(DSS) and FMT(DSS + DHA) mice. Data are presented as mean ± SD (*n* = 3 mice/group). P values were calculated using an unpaired t test. *P < 0.05, **P < 0.01

We next assessed whether DHA influenced mucosal immune regulation. DHA treatment significantly increased the frequency of colonic Treg cells, defined as CD25 + Foxp3+ cells, compared with DSS treatment alone (4.24% vs. 2.66%; *P* < 0.05; Fig. 6A). However, ABX-mediated microbiota depletion abolished this effect, as Treg percentages did not differ significantly between the ABX(DSS) and ABX(DSS + DHA) groups (3.64% vs. 3.52%; *P* = 0.5875; Fig. 6B). Conversely, recipients of FMT from DHA-treated donors showed a higher Treg frequency than recipients of FMT from DSS-treated donors (5.23% vs. 1.69%; *P* < 0.05; Fig. 6C). These findings demonstrate that DHA expands colonic Treg cell populations through a microbiota-dependent mechanism.

## Discussion

In the present study, we demonstrated that DHA markedly alleviated DSS-induced colitis in mice. DHA reduced body weight loss, mortality, DAI scores, colon shortening, histological damage, and pro-inflammatory cytokine expression. Mechanistically, ABX and FMT experiments showed that the protective effects of DHA depended largely on the gut microbiota. DHA reshaped the gut microbial community, improved intestinal barrier integrity, and increased the frequency of colonic Treg cells. These findings support a model in which DHA mitigates experimental colitis by modulating microbiota-barrier-immune interactions.

Omega-3 PUFAs are essential fatty acids that must be obtained from dietary sources. A growing body of evidence indicates that n-3 PUFAs exert anti-inflammatory effects, including in the context of IBD (Ajabnoor et al. 2021; Charpentier et al. 2018; Mozaffari et al. 2020). Epidemiological studies have suggested that high intake of n-6 PUFAs and low intake of n-3 PUFAs are associated with increased UC risk (Scaioli et al. 2017). Mendelian randomization and cohort-based analyses have also supported a protective association between n-3 PUFA status and IBD or UC risk (Astore et al. 2022; He et al. 2022). Our results extend these observations by showing that oral DHA administration exerts protective effects in a DSS-induced colitis model, as evidenced by improved clinical, histological, and inflammatory outcomes.

Gut microbiota dysbiosis is a major feature of UC and contributes to disease progression by altering microbial composition, microbial metabolites, epithelial barrier function, and immune responses (Franzosa et al. 2019; Pittayanon et al. 2020; Hu et al. 2021). Restoring microbial balance has been shown to alleviate DSS-induced colitis in experimental models (Dong et al. 2021; Dou et al. 2020). In this study, microbiota depletion abolished the protective effects of DHA, whereas FMT from DHA-treated donors transferred protection to recipient mice. These findings provide functional evidence that the gut microbiota is not merely associated with DHA-mediated protection but is required for the anti-colitic effects of DHA.

DHA also significantly altered the diversity and composition of the gut microbiota. In particular, DHA increased the relative abundance of *Bifidobacterium*, a genus frequently associated with intestinal health. Previous studies have shown that *Bifidobacterium longum* and *Bifidobacterium bifidum* can alleviate experimental colitis, enhance epithelial barrier function, and suppress inflammatory responses (Cui et al. 2022; Yao et al. 2021). Therefore, enrichment of *Bifidobacterium* may contribute to the protective effect of DHA in DSS-induced colitis. However, whether *Bifidobacterium* directly mediates the observed phenotype requires further validation using bacterial isolation, mono-colonization, or targeted supplementation experiments.

Impairment of the intestinal epithelial barrier is a critical event in UC pathogenesis (Miner-Williams and Moughan 2016; Salim and Soderholm 2011). Barrier dysfunction increases epithelial permeability and facilitates the translocation of luminal antigens, toxins, and microorganisms into the mucosa, thereby amplifying inflammation (van der Post et al. 2019). Tight junction proteins such as occludin and ZO-1 are essential for maintaining epithelial integrity and regulating intestinal permeability (Kuo et al. 2021; Merga et al. 2014; Suzuki 2013). In our study, DHA reduced serum FITC-dextran levels and restored occludin and ZO-1 expression in DSS-treated mice, indicating improved intestinal barrier function. Importantly, this barrier-protective effect was lost after ABX treatment but was reproduced by FMT from DHA-treated donors, further supporting a microbiota-dependent mechanism.

Mucosal immune homeostasis is shaped by dynamic interactions between the gut microbiota and host immune system (Honda and Littman 2016). Treg cells are central regulators of intestinal immune tolerance and suppress excessive effector T-cell activation and pro-inflammatory cytokine production (Clough et al. 2020). Alterations in Treg cell abundance or function may contribute to IBD development and progression (Britton et al. 2019). We found that DHA increased the frequency of colonic Treg cells, whereas this effect was abolished by ABX-mediated microbiota depletion and restored by FMT from DHA-treated donors. These findings suggest that DHA promotes mucosal immune regulation through microbiota-dependent expansion of Treg cells. In parallel, DHA reduced DSS-induced expression of IL-1β, IL-6, and TNF-α, indicating suppression of colonic pro-inflammatory responses.

This study has several limitations. First, although DHA increased the abundance of several beneficial taxa, the specific bacterial mediators responsible for its protective effects remain to be identified. Second, microbial metabolites that may link DHA-induced microbiota remodeling to barrier repair and Treg expansion were not examined. Third, this study was performed in an acute DSS-induced colitis model; therefore, the efficacy of DHA should be further validated in chronic colitis models and, ultimately, in clinical studies. Future work should integrate metagenomic, metabolomic, and targeted bacterial intervention approaches to define the precise microbiota-derived mechanisms underlying DHA-mediated protection.

## Conclusions

**Fig. 7:**
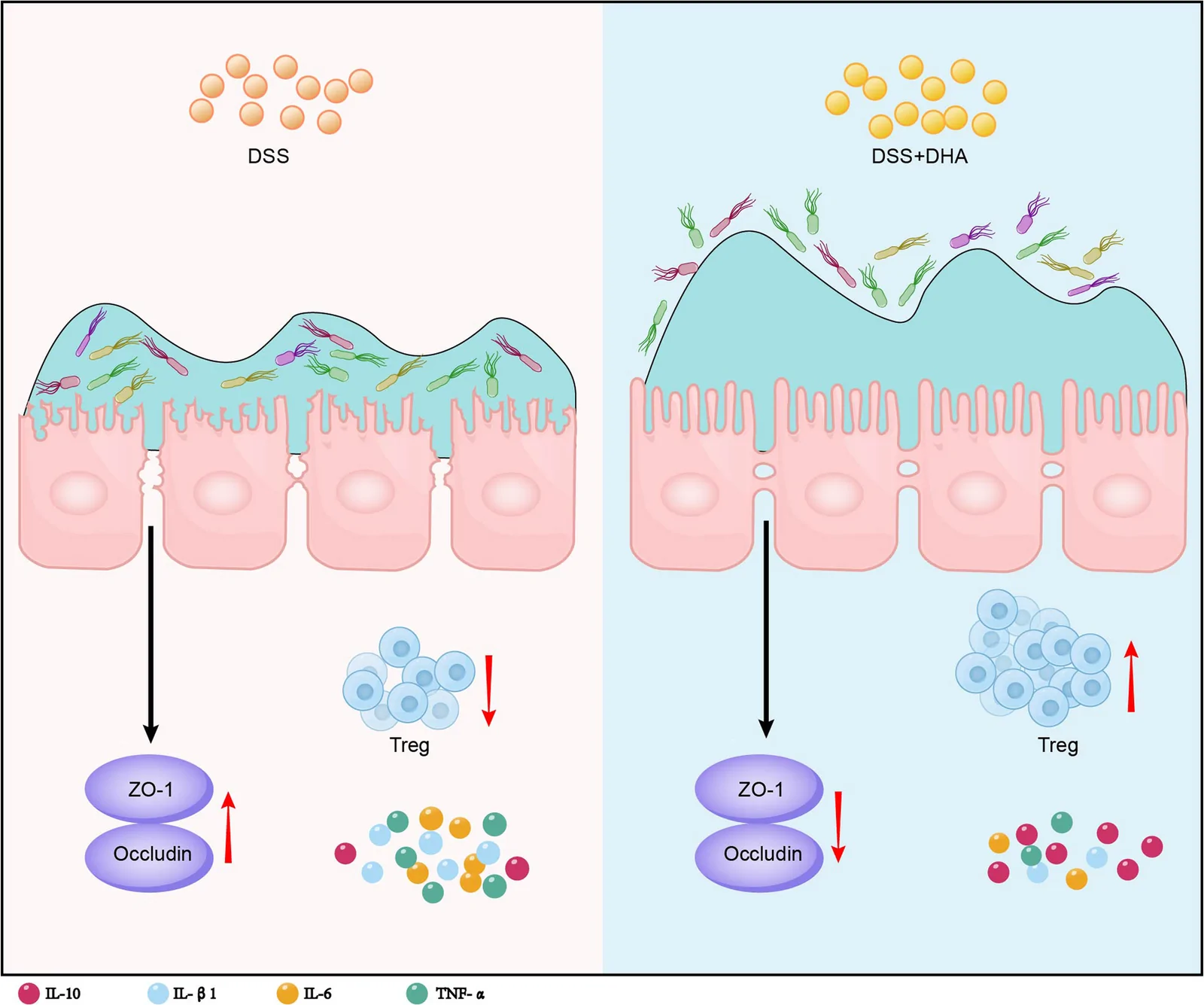
Schematic illustration of the protective effects of DHA in ulcerative colitis. DHA alleviates DSS-induced colitis by modulating the gut microbiota, enhancing tight junction protein expression, preserving intestinal barrier integrity, and suppressing inflammatory cell infiltration

In conclusion, DHA alleviates experimental colitis by reshaping the gut microbiota, restoring intestinal barrier integrity, and regulating mucosal immune homeostasis, particularly through microbiota-dependent mechanisms (Fig. 7). These findings suggest that DHA has potential as a dietary supplement or adjunctive therapeutic strategy for UC. Future studies should identify the specific microbial taxa and metabolites responsible for the beneficial effects of DHA and optimize its translational application in intestinal inflammatory disorders.

## Supplementary Information

Below is the link to the electronic supplementary material.

Supplementary Material 1 (DOCX 16.5 KB)

## Data Availability

No datasets were generated or analysed during the current study.

## Abbreviations

ABX: antibiotic cocktail
ANOVA: analysis of variance
CD: Crohn’s disease
DAB: 3,3’-diaminobenzidine
DAI: disease activity index
DHA: docosahexaenoic acid
DSS: dextran sulfate sodium
FBS: fetal bovine serum
FITC: fluorescein isothiocyanate
FMT: fecal microbiota transplantation
H&E: hematoxylin and eosin
IBD: inflammatory bowel disease
IL: interleukin
LDA: linear discriminant analysis
LEfSe: linear discriminant analysis effect size
OTU: operational taxonomic unit
PBS: phosphate-buffered saline
PCoA: principal coordinate analysis
PUFA: polyunsaturated fatty acid
qRT-PCR: quantitative real-time polymerase chain reaction
SASP: sulfasalazine
SD: standard deviation
TJ: tight junction
TNF-α: tumor necrosis factor-α
Treg: regulatory T cell
UC: ulcerative colitis
ZO-1: zonula occludens-1
