# Supplementary material for: Docosahexaenoic acid alleviates DSS-induced colitis by regulating the gut microbiota and restoring the gut barrier
Source: Int Microbiol. 2026 Jul 3;29(7):1451–63. doi: 10.1007/s10123-026-00859-4 (PMC13653849; doi:10.1007/s10123-026-00859-4)
Supplement: Supplementary file 1 [file 10123_2026_859_MOESM1_ESM.docx]

Supplemental Table 1. Primers for qRT-PCR detection.

| Gene | F（5’-3’） | R（5’-3’） |
| --- | --- | --- |
| *IL-1β* | TGCCACCTTTTGACAGTGATG | TGATGTGCTGCTGCGAGATT |
| *IL-6* | CTGCAAGAGACTTCCATCCAG | AGTGGTATAGACAGGTCTGTTGG |
| *TNFα* | CCACCACGCTCTTCTGTCTAC | AGGGTCTGGGCCATAGAACT |
| *Actin* | CGTGAAAAGATGACCCAGATCA | CACAGCCTGGATGGCTACGT |
